# Evolutionary ecophysiology in extreme environments under a global change scenario

**DOI:** 10.1093/conphys/coaf059

**Published:** 2025-08-11

**Authors:** Pablo Burraco, Lucy Hawkes, Natalie Pilakouta, Frédéric Angelier, Kristien I Brans, Germán Orizaola

**Affiliations:** Doñana Biological Station (CSIC), c/ Americo Vespucio 26, 41092 Seville, Spain; Department of Biosciences, University of Exeter, Hatherly Laboratories, Prince of Wales Road, Exeter EX4 4PS, UK; Centre for Biological Diversity, Biomedical Sciences Research Complex, University of St Andrews, Saint Andrews KY16 9TZ, UK; Centre d’Études Biologiques de Chizé, CNRS—La Rochelle Université, UMR7372, 405 Route de Prissé la Charrière, 79360 Villiers-en-Bois, France; Department of Biology, Vrije Universiteit Brussel, Etterbeek Brussels, Pleinlaan 2, 1050 Ixelles, Belgium; IMIB-Biodiversity Research Institute (Univ. Oviedo-CSIC-Princip. Asturias), Campus de Mieres, Edificio de Investigación – 5ª planta, c/ Gonzalo Gutiérrez Quirós s/n, 33600 Mieres, Asturias, Spain; Zoology Unit, Department of Biology of Organisms and Systems, University of Oviedo, Oviedo, c/ Catedrático Rodrigo Uría s/n, 33071, Asturias, Spain

**Keywords:** Anthropogenic disturbances, biomarkers, climate change, conservation physiology, environmental stress

## Abstract

As wildlife increasingly has to face levels of environmental conditions that go far beyond normal ranges, understanding the ecological and evolutionary dynamics behind such extreme scenarios becomes essential for animal conservation. Here, we discuss the eco-physiological singularities of wildlife coping with extreme conditions. We first discuss the conditions under which scenarios can be considered ‘extreme’. This includes distinguishing the nature of natural and anthropogenic disturbances, considering aspects such as their intensities, as well as the understanding of species biology and evolutionary history. To exemplify the diversity of wildlife responses to extreme conditions, we highlight five different representative study cases (two with natural causes, three of anthropogenic origin): birds at high altitude, fish in geothermal habitats, birds in pesticide-laden farmlands, invertebrates in urban ponds, and amphibians in radioactive zones. These examples illustrate the diverse physiological and ecological responses to extreme factors, emphasizing the complexity of wildlife adaptation under different scenarios. However, they also reveal significant knowledge gaps regarding long-term effects of responses to extreme environments, and the mechanistic basis behind these processes. Future research should ideally include long-term approaches making use of validated physiological markers of individual, population or species health or fitness. This information could be then incorporated into mechanistic models like Species Distribution Models (SDMs) to predict species geographic occurrence and the impact of future extreme scenarios. Such holistic and integrative physiological approaches will enhance our understanding of species and population resilience, and will facilitate the identification of vulnerable populations, ultimately improving management strategies. By prioritizing these research efforts, we will better anticipate the impacts of environmental changes on wildlife health, and thus improve biodiversity conservation strategies.

## Introduction

The study of wildlife responses to extreme environments is key to understand eco-evolutionary dynamics under the current context of global changes (Alberti, 2015, 2024; Fouqueau and Polechová, 2024). A classic definition of ‘extreme environment’ refers to a condition detrimental or fatal to highly organized life forms (Thiel, 2011). This view is often linked to extremophile organisms, such as microbes, which thrive in conditions beyond the tolerance limits of most other taxa (e.g. hydrothermal vents, highly acidic waters, or soda lakes; Rothschild and Mancinelli, 2001). Organisms capable of surviving these conditions often develop life-history or physiological strategies to cope with the stress associated with such environments, while balancing costs such as reductions in stored resources or loss of fitness (Wingfield *et al.*, 2011; Boothby, 2019; Ando *et al.*, 2021).

A broader interpretation of extreme environments should consider that most environmental factors naturally occur on a continuum (Rothschild and Mancinelli, 2001; Hardie and Hutchings, 2010; Wingfield *et al.*, 2011). Thus, marginal and infrequent values outside the bounds of normal variability (e.g. the interquartile or >90% range of values for each condition within a given ecosystem) could be defined as ‘extreme’ (Rothschild and Mancinelli, 2001). These ‘severe conditions’ (sensu Wingfield *et al.*, 2011) may be far from life-limiting but can compromise the normal functioning of organisms. From an evolutionary standpoint, *extreme* or *severe* environments can include high selective pressures, which may lead to reductions in fitness. In this context, species biology and distribution should be the two main aspects used to define environmental extremeness. For instance, the impact of a given condition can even vary within the same species due to factors, such as life-stage–dependent responses or local adaptations (e.g. Austin and Moehring, 2019; Kelly, 2019; Vicuña *et al.*, 2019; Madeira *et al.*, 2020; Ubach *et al.*, 2022). Likewise, the occurrence and persistence of certain conditions can influence the responses of organisms. In some cases, exposure to ‘extreme’ conditions can be permanent (e.g. low oxygen levels at extreme altitude), whereas in others, it can be only transient (e.g. high temperatures during a heat-wave event). Among the former, many natural environmental conditions have been historically classified as extreme in the literature, including low temperatures in the polar regions or aridity in deserts. However, in most of these natural extreme environments, many taxa have developed physiological and life-history adaptations (Whiteman, 2021; Storz *et al.*, 2023; e.g. polar bears, freezing amphibians, mice in high mountains; Schmidt *et al.*, 2024). Hence, the occurrence, intensity, and persistence of a given condition, along with the adaptive potential (evolutionary history) of organisms, will determine whether a condition or combination of conditions, can be considered as ‘extreme’ for an individual, population or species.

Human actions have modified not only the range, frequency and intensity of natural conditions (e.g. temperature, precipitation, light) but have also introduced novel sources of disturbance such as the release of chemical pollutants (e.g. pesticides, herbicides, heavy metals; Fischer *et al.*, 2013; Saaristo *et al.*, 2018). These anthropogenic conditions can impose physiological challenges, either on their own or synergistically with conditions that were previously not considered as extreme, ultimately leading to detrimental consequences. Yet, evidence suggests that new extreme environments can also drive phenotypic plasticity evolution, trait novelty and rapid adaptation (Chevin and Hoffmann, 2017; Wang and Guo, 2019; Parrilli *et al.*, 2021). The understanding of the machinery underlying such evolutionary responses to extreme environments is therefore a major challenge for evolutionary biologists, ecologists and conservationists (Storey and Storey, 2005; Madliger *et al.*, 2018; Gunga, 2020). This knowledge is crucial to predict biotic responses to future global change scenarios and to develop effective conservation actions.

Research on wildlife responses to natural and anthropogenic extreme environments has made great advances in the last years. This progress includes a deeper understanding of the biology and physiology of different study species or populations, a knowledge that is increasingly achieved through more complex, multifactorial, and realistic field and/or laboratory-based approaches (e.g. Maček *et al.*, 2016; Dawson *et al.*, 2020; Burraco *et al.*, 2021c; Gerhard *et al.*, 2023; see below details of some study systems). Long-term and longitudinal studies have shed light on how extreme factors impact individual condition and fitness, then contributing to understand the eco-evolutionary consequences of certain extreme environments (Hoset *et al.*, 2014; Jenouvrier *et al.*, 2015; e.g. Barneche *et al.*, 2021; Gicquel *et al.*, 2022). However, the study of ‘broad sense’ extreme environments is a relatively new field within a conservation framework, and therefore, there is a wide range of challenges and future directions to explore (Bailey and van de Pol, 2016; Van de Pol *et al.*, 2017). In this paper, we discuss physiological and eco-evolutionary singularities of different extreme scenarios, addressing both methodological and empirical aspects. We provide five distinct examples of research conducted on wildlife coping with different extreme conditions of natural or anthropogenic origin. For each case, we summarize the main characteristics of each extreme condition and its effects on the ecology, evolution and conservation of the study organisms, while examining the methodological challenges and unique features of each system. Finally, we offer some recommendations that could be relevant for future research in the field, with emphasis on the integration of physiological information to improve conservation and restoration actions.

## Case Studies of Ecophysiological Approaches to Extreme Environments

While the number of studies addressing the eco-physiological consequences of extreme environments is rapidly increasing, much of the research is scattered across systems and taxa, making it challenging to form general conclusions. Nonetheless, a substantial body of work documents physiological adjustments and evolutionary responses to a wide array of stressors in both natural and human-altered settings. For example, desert marsupials such as the wallabies have evolved exceptionally efficient renal physiology to cope with chronic water scarcity (Bradshaw *et al.*, 2001), whereas intertidal mussels use heat-shock proteins and dynamic shell gaping to withstand episodes of extremely high temperatures during low tide (Buckley *et al.*, 2001). Antarctic and Artic fish convergently produce antifreeze glycoproteins that prevent ice-crystal growth at sub-zero sea temperatures (Chen *et al.*, 1997), and reef-building corals in naturally acidic CO₂ vents show trans-generational shifts in calcification strategies that confer partial tolerance to ocean acidification (Kurihara *et al.*, 2020). Pants also provide compelling evidence: metal-hyperaccumulator species like *Arabidopsis halleri* thrive on heavy-metal substrates by up-regulating metal-chelation pathways (Noor *et al.*, 2024). Collectively, these and many other studies illustrate convergent themes—enhanced cellular repair, metabolic re-engineering and novel protective compounds—that underpin wildlife resilience when environmental conditions move beyond historical baselines.

**Figure 1:**
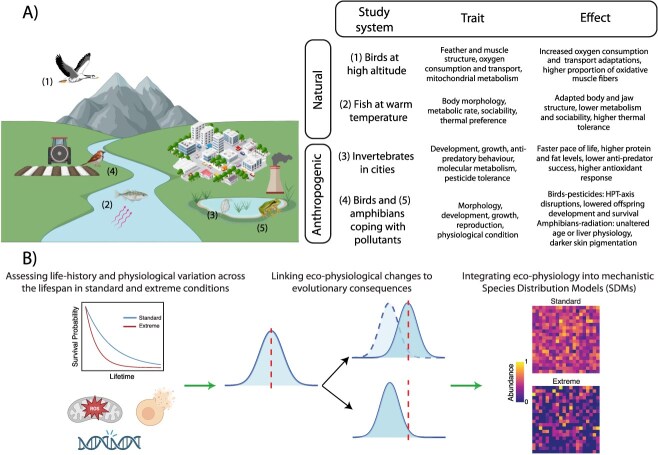
(A) The left section illustrates different examples of extreme environmental conditions—both of natural and anthropogenic origin—that wildlife may face. The right section summarizes previous research on various study systems, including birds living at high altitudes, fish inhabiting environments with extreme temperatures (e.g. geothermal habitats), invertebrates in urbanized areas, and vertebrates exposed to high levels of chemical pollution (e.g. birds in pesticide-contaminated farmlands, and amphibians in radioactive areas). For each system, key traits and the main observed effects are highlighted. (B) A conceptual overview of how eco-physiological research can be applied to understand and predict the consequences of environmental extremes for wildlife. (Left) This type of approach first requires characterizing normal patterns in life-history traits (e.g. lifespan, fitness) and physiological health markers (e.g. oxidative stress, DNA damage, cell apoptosis). (Middle) By studying shifts in these parameters, we can validate biomarkers and mechanistically unravel the eco-evolutionary consequences of environmental extremes. (Right) Finally, this information can then be integrated into mechanistic Species Distribution Models (SDMs) to improve predictions of environmental change impacts on biodiversity and to inform more effective conservation and restoration planning. Created with BioRender.com.

A comprehensive evaluation of how animals shape their physiology to cope with extreme environments, either of natural and anthropogenic origin, is crucial to mechanistically understanding the eco-evolutionary consequences underlying some ecosystems. To show and discuss some possible approaches in this direction, we present five examples of research programs investigating the physiological effects and adaptations of wildlife exposed to extreme environments (Fig. 1A). Two of these examples focus on natural extremes (i.e. low oxygen at high altitudes, and elevated temperatures in geothermally warmed lakes), while the other three address anthropogenic extremes (i.e. urbanization, pesticides in agricultural fields, and ionizing radiation following a nuclear accident).

### Birds at extreme altitude

The highest parts of the planet such as the Tibetan Plateau in Asia, the Ethiopian highlands in Africa and the Andean Altiplano in South America, experience seasonally from extreme hypoxia, cold and xeric conditions and pose complex challenges to biodiversity (da Costa *et al.*, 2023). Despite this, birds are unparalleled in the animal kingdom for making spectacular migrations across vast scales, including travelling over the highest regions on earth. Flight is a particularly demanding form of exercise, and the pulmonary system of birds has evolved into an unidirectionally ventilated system with cross-current oxygen extraction, which permits huge rates of oxygen consumption (approximately twice as large as mammals; Butler *et al.*, 1988; Maina, 2008; Cieri and Farmer, 2016). Both of these aspects of the evolutionary ecology and physiology of birds has meant that birds are particularly well adapted to obtaining oxygen in hypoxic environments, not only at rest, but also during exercise there at high rates of volume of oxygen (V$\mathrm{O}$_2_; Maina, 2008; Cieri and Farmer, 2016). Although a range of work has demonstrated adaptations to high altitude in species such as mice (Chen *et al.*, 2023; Storz and Scott, 2023), a classic study system has been the bar-headed goose *Anser indicus* (Fig. 1A; Butler, 2010; Scott *et al.*, 2015; Hawkes *et al.*, 2017). In 1953, the expedition led by Tenzing Norgay and Edmund Hillary claimed to see bar-headed geese flying over Mount Everet (Swan, 1970). Since then, adaptations throughout the oxygen transport cascade have been extensively studied (Butler, 2010; Scott *et al.*, 2015; Hawkes *et al.*, 2017).

Geese are relatively tractable subjects to study, being large bodied and robust. To date, bar-headed geese have been studied in tissue preparations (Scott *et al.*, 2009), at rest in captivity (Black and Tenney, 1980; Scott and Milsom, 2007), running on a treadmill (Black and Tenney, 1980; Hawkes *et al.*, 2014), flying in a wind tunnel (Ward *et al.*, 2002; Meir and Milsom, 2013) and in the wild (Hawkes *et al.*, 2013; Scott *et al.*, 2015). These studies have been able to make use of approaches from classic physiological techniques (e.g. haemoglobin-oxygen dissociation curves) to state-of-the-art biotelemetry devices (Scott *et al.*, 2015).

Owing to the cardiovascular adaptations of birds to fund extremely high rates of oxygen consumption, most bird species can likely cope better than other taxa at high altitudes. Downy feathers can also help birds to cope with extreme cold (Terrill and Shultz, 2023), and extremely dry conditions may pose surprisingly little challenge to birds, because they can catabolize flight muscle during flight, producing metabolic water (Klaassen, 1996). It appears currently that few species of birds are as tolerant of hypoxia as the bar-headed goose, which has adaptations at every step of the oxygen transport cascade (Butler, 2010; Scott *et al.*, 2015; Hawkes *et al.*, 2017), but because flight costs scale following a power law with body mass (Bishop, 1997), it is likely that smaller species (e.g. alpine chough *Pyrrhocorax graculus digitatus*, or other smaller passerine species) may have proportionally lower costs, and therefore superior performance at altitude to even the bar-headed goose.

The ability of birds to perform well, even in hypoxic, cold and xeric conditions has important ramifications that warrant future study. First, birds can be major vectors for viruses, such as bird flu (Caliendo *et al.*, 2022; Cui *et al.*, 2022; Ramey *et al.*, 2022; Klaassen and Wille, 2023; Lane *et al.*, 2024), dispersing agents over long distances. Extreme environments normally inhibit pathogen survival, and/or restrict the distribution of their vectors, but species that can cope well with extreme environments can disperse pathogens through such environments, in some cases, even if asymptomatically infected (Eikenaar and Hegemann, 2016; Eikenaar *et al.*, 2020; Tobolka *et al.*, 2024). Second, as more species move into high altitude environments seeking climate refugia (Keppel *et al.*, 2024), birds will come into contact with a wider range of species, with differing abilities to compete for limited resources (Stenseth *et al.*, 2015). Finally, the ability for vertebrate species to cope with extreme oxygen conditions continues to be of interest to medicine, sport and space travel (Keil *et al.*, 2015; Magalhães, 2015).

### Fish in geothermal environments

Geothermal environments are key not only for the study of early life evolution on Earth but also for examining adaptation to extreme temperatures in wildlife (Jutfelt, 2020). For example, previous studies have taken advantage of a high-elevation geothermal stream system in the Andes to study the effects of temperature on fish body size, macroinvertebrate community composition and transcriptional plasticity in anurans (e.g. Pastenes *et al.*, 2017; Quenta-Herrera *et al.*, 2021). There are also a number of studies on the reproductive phenology, thermal physiology and temperature preferences of ‘hot spring frogs’ (*Buergeria japonica*) found in geothermal hot springs in Taiwan and Japan (e.g. Chen *et al.*, 2001; Komaki *et al.*, 2020).

Geothermally warmed water bodies (also known as ‘hot springs’) adjacent to ambient-temperature lakes, which are much colder (Pilakouta *et al.*, 2020, 2023a), are abundant in Iceland. There is one fish species that can be found in both of these thermal habitats: the threespine stickleback *Gasterosteus aculeatus* (Fig. 1A). These stickleback populations have been exposed to contrasting thermal regimes for hundreds to thousands of generations, and there are no differences in water chemistry between these thermal habitats other than temperature (Pilakouta *et al.*, 2020). Given these repeated and independent pairs of warm and cold populations in close proximity, we can use the power of evolutionary replication to test the predictability of adaptation to high temperature by testing for parallel evolution across these population pairs.

A major advantage of this study system is the small geographic scale of these population pairs, which allows us to avoid confounding factors associated with latitudinal or altitudinal comparisons of different thermal environments (Jutfelt, 2020). Threespine sticklebacks are an established model in ecology and evolutionary biology with a well-annotated genome and transcriptome, and well-developed protocols for rearing and breeding this species in the lab (Reid *et al.*, 2021). Thus, in addition to identifying phenotypic differences in wild-caught fish from warm and cold populations, we can conduct common-garden or reciprocal-transplant experiments to disentangle plastic and evolutionary changes in response to temperature in these populations (e.g. Pilakouta *et al.*, 2023c). We can then link these phenotypic changes to the underlying molecular mechanisms and their effects on ecosystem-level processes.

Work on this study system has shown that hot-spring fish have diverged morphologically, physiologically and behaviourally compared to fish in ambient-temperature lakes. Firstly, fish living in hot springs are deeper-bodied, have shorter jaws and steeper craniofacial profiles, reflecting a more benthic diet (Pilakouta *et al.*, 2023a). Secondly, they have a lower metabolic rate (Pilakouta *et al.*, 2020), which could be adaptive, especially during the winter, when hot-spring fish are still experiencing relatively high temperatures but food, such as insect larvae, is scarce. Hot-spring fish are also less social (Pilakouta *et al.*, 2023c). Lower sociability can be adaptive when there is high competition for food (Webster and Hart, 2006), as may be the case in these warm habitats. Lastly, fish from warm habitats prefer cooler temperatures when given a choice in the lab, even though they can persist at much higher temperatures in the wild (Pilakouta *et al.*, 2023b).

This kind of natural experiments can revolutionize our understanding of how organisms adapt to high temperatures, with still many important questions to address in this study system. For example, it would be worthwhile to further investigate physiological adaptation in response to food limitation in hot springs. A study on cavefish, which experience long periods of food deprivation, found evidence for insulin resistance as an adaptation to cope with their nutrient-limited environment (Riddle *et al.*, 2018). Similarly, preliminary data show that hot-spring fish exhibit a number of characteristics associated with diabetes, with respect to their insulin resistance, fat storage, appetite and starvation resistance (N. Pilakouta, unpublished data). Using a wild organism where the symptoms of diabetes may have an adaptive purpose can overcome many of the limitations of current laboratory models (Albertson *et al.*, 2009). Thus, linking this widely used evolutionary model system to a human disease has potential clinical applications, for example by allowing us to identify novel biomarkers or genetic pathways associated with glucose metabolism that could be applied to humans (Albertson *et al.*, 2009; Riddle *et al.*, 2018; Krishnan *et al.*, 2022; Xiong *et al.*, 2022).

### Invertebrates in urban environments

Urban abiotic and biotic environments are drastically altered via reduced vegetation cover and habitat fragmentation, elevated pollution levels of various sorts (e.g. thermal, chemical, light, noise pollution), the introduction of non-native species, telecoupling and the fingerprint of human societal vectors at play (i.e. economics, technological development, culture, politics). Throughout the years, cities and urbanized areas have therefore been identified as uniquely complex ecosystems, which shape ecology (Grimm *et al.*, 2008) and evolution (Szulkin *et al.*, 2020), and now set the stage for potential profound specific socio-eco-evolutionary feedback (Des Roches *et al.*, 2021; Alberti, 2024). While ponds and pools are vital for biodiversity (Hill *et al.*, 2021) and the residing biota is hypothesized to be more severely impacted by joint urban perturbations given their closed nature (Oertli and Parris, 2019), they remained largely unexplored in cities for long.

Experimental work using field and common garden studies in the strongly urbanized region of Flanders (Belgium) tested for urban adaptive evolutionary trait change in the cladoceran *Daphnia magna* (Fig. 1A), and examined potential eco-evolutionary feedback, focusing on the context of urban warming and chemical pollution (Brans *et al.*, 2017, 2018b, 2021). *Daphnia magna* plays a dual key role in freshwater food webs, being a food source for higher trophic levels, as well as exerting top-down control on phytoplankton thereby stabilizing clear water phases and preventing toxic bloom formation (Ebert, 2022). *Daphnia magna* is cyclical parthenogenetic, which enables to culture asexually replicated isoclonal lineages for multiple generations prior to trait assessment in common garden experiments, thus reducing the impact of (grand)maternal effects (Ebert, 2022). First, by studying replicated urban and rural populations, it was shown that *D. magna* exhibits genetic differentiation for key traits related to thermal adaptation to the observed warming in city ponds in the study region (i.e. the Urban Hot Tub Effect; Brans *et al.*, 2018a); urban populations evolved a higher heat tolerance, smaller size at maturity, higher haemoglobin tissue content (Brans *et al.*, 2017) and a faster pace-of life, including faster development and maturation, as well as higher fecundity, compared to rural populations (Brans *et al.*, 2018b). Physiological analyses on body tissue, revealed urban populations having higher storage levels of proteins, fat and carbohydrates and a more optimized antioxidant defence system. These physiological characteristics were intertwined with life history via the evolution of a pace-of-life syndrome in urban, but not rural animals (Brans *et al.*, 2018b). Finally, evolved higher organophosphate pesticide tolerance was detected in urban compared to rural populations by studying survival responses to chlorpyrifos, a commonly used pesticide in urban environments in the region till 2020 (Brans *et al.*, 2021). Remarkably, an extended study including water fleas and damselfly nymphs (Coenagrionidae), common water flea predators for which similar urban evolutionary responses have been documented (Tüzün *et al.*, 2017), found a cryptic thermal eco-evolutionary feedback. More specifically, urban evolution in water fleas offsets the evolved higher predation rates observed in urban compared to rural damselfly nymphs during a simulated heatwave (Vanvelk *et al.*, 2021; Brans *et al.*, 2022).

While *D. magna* reproduction cycles are key in assessing contributions of genetic differentiation, plasticity and evolution of plasticity (Govaert *et al.*, 2016; Ebert, 2022), such laboratory quantitative genetic analyses and settings only limitedly mimic *in situ* environmental conditions. In order to asses effective evo-to-socio feedback in the light of, for example, effective top-down control of noxious algal and cyanobacterial blooms in cities, future experiments would benefit from a common gardening reciprocal transplant set-up (De Meester *et al.*, 2019; Brans *et al.*, 2020). This enables to test whether the urban evolutionary background of *D. magna* shapes ecological food web interactions and ultimately bloom formation. In addition, there is growing evidence that the *Daphnia* gut microbiomes plays an important role in acclimation and adaptation to toxic cyanobacteria (Houwenhuyse *et al.*, 2021). To what extent urban *D. magna* are characterized by a distinct gut microbiome, and whether this drives adaptive and acclimation responses to urbanization-related stressors, as well as toxic cyanobacteria, is unclear. Thus, a reciprocal transplant across urban and rural settings enables to shed light on how evolutionary trait change in a key freshwater species or organism, can mitigate negative impacts of warming and eutrophication, two key drivers of a shift to a turbid, cyanobacteria-dominated state of freshwater ecosystems, and, importantly, predicted to be the most pronounced threats to freshwater ecosystems worldwide under future climate change.

### Birds in polluted agricultural fields

Many wild vertebrate species have co-evolved with human activities for decades or even centuries. As a result, some of them are particularly well adapted to human activities, and this allowed them to thrive in anthropogenic environments by occupying specific ecological niches that were created by humans (Hendry *et al.*, 2017). Farmland birds represent very good examples of this phenomenon as they mainly rely on historical agricultural activities. However, agricultural practices have changed at an unprecedent rate since the middle of the 20th century. Mechanization, increase in the size of land plots and the alteration of many ecological infrastructures (e.g. woods, hedgerows, ponds, etc.) have resulted in drastic environmental changes that have imposed new, and often, extreme constraints to farmland birds (Stanton *et al.*, 2018; Rigal *et al.*, 2023). Among them, the massive use of pesticides has represented a real threat to wild birds with huge impact on some species that were at the edge of extinction (Carson, 1962). Although several ‘old’ historical pesticides have been banned in several countries (e.g. DDT), it is not true in all countries. When banned, they have progressively and continuously been replaced by new organic molecules that aimed to either better control pests or improve health safety (Matthews, 2015). These pesticides are widely used in multiple agroecosystems worldwide, but vineyards often represent one of the agroecosystems that rely the most on pesticides and especially organic fungicides, such as triazoles (Komárek *et al.*, 2010; García *et al.*, 2022), except in a few specific cases (e.g. organic vineyards). Indeed, grapes are very sensitive to pest fungi and the use of fungicides aims to improve grape yields, especially under cold and wet climate (Geppert *et al.*, 2024).

Over the last decade, field and laboratory studies have been conducted to assess the physiological and ecological consequences of living in extremely highly contaminated vineyards. By comparing the contamination levels of birds living in vineyards, other agroecosystems, and theoretically uncontaminated areas, studies aimed to test whether vineyards represent an extreme environment regarding the use of triazoles (Angelier *et al.*, 2023; Prouteau *et al.*, 2025). By using experimental setups, these studies also aimed to mimic the contamination that can be found in vineyards and to test its impact on organismal systems and performance in farmland birds.

Overall, birds living in vineyards show high levels of some contaminants, notably triazoles, the main organic fungicides used in crops (Angelier *et al.*, 2023; Fig. 1A). This contamination seems ubiquitous in agroecosystems because birds were contaminated in locations close to cereal crops (although to a much lower level). Studies using experimental approaches showed that such extreme contamination by triazoles in vineyards can induce some alterations in numerous and key organismal systems (reviewed in Jiménez-Peñuela *et al.*, 2024), such as metabolism (Bellot *et al.*, 2022), immunity (Fernández-Vizcaíno *et al.*, 2024) and the functioning of the hypothalamus-pituitary-thyroid axis (Bellot *et al.*, 2025a). These physiological perturbations can alter the development and the survival of offspring (Bellot *et al.*, 2025b), echoing the reported dramatic drop of farmland birds in the last decades (i.e. from 1980 to 2016, Rigal *et al.*, 2023). Importantly, these results are not limited to a few study species (house sparrow, *Passer domesticus*, European blackbird, *Turdus merula*), as other articles have convincingly demonstrated detrimental impacts of these triazole fungicides on the reproduction and the physiology of other farmland species, such as red-legged partridges, *Alectoris rufa* (Lopez-Antia *et al.*, 2021; e.g. Fernández-Vizcaíno *et al.*, 2024). Altogether, these results demonstrate that vineyards certainly represent an extreme environment for farmland birds. The continuous release of new triazole substances, combined with the massive use of these fungicides in vineyards, may limit the ability of farmland birds to adapt quickly enough to this anthropogenic selection pressure and might therefore jeopardize bird biodiversity in vineyards (Zielonka *et al.*, 2024).

Multiple recent articles have demonstrated that vineyards are associated with the massive use of multiple fungicides including triazoles (Komárek *et al.*, 2010; e.g. García *et al.*, 2022), and that they can probably affect the fitness of farmland birds (reviewed in Jiménez-Peñuela *et al.*, 2024). However, we definitely lack data to better understand how these birds may have evolved some mechanisms to counteract the detrimental effects of these pesticides on their performance (survival and reproduction). Future studies focusing on inter-population sensitivity to these pesticides are now warranted to understand if birds could adapt to such extreme contamination.

### Amphibians in radioactive areas

Life has evolved under various sources of ionizing radiation, originated mostly from naturally occurring radioactive materials in Earth’s crust or from cosmic rays (i.e. background ionizing radiation; Sohrabi, 2013). The development of nuclear technology, especially since the second half of 20th century, lead to the industrial use of radioactive isotopes for the production of energy, the design of nuclear weapons, and the development of nuclear medicine, among many other uses (IAEA, 2024). As a consequence of both deliberate actions (e.g. use and test of nuclear weapons) or unintentional releases of radioactive material (e.g. nuclear accidents), the increase of radioactive levels in some areas may pose risks to the environment (e.g. Beresford and Copplestone, 2011), and it represents an example of new extreme conditions experienced by wildlife.

Ionizing radiation released during the decay of radioactive isotopes can directly impact wildlife by damaging DNA strands through physical breaks in nucleotide sequences, or indirectly by interacting with other molecules (mostly water) which generates free radicals that can compromise individual performance and survival (Santivasi and Xia, 2014). Some of these effects have been studied in detail in areas that experienced severe accidents in nuclear power plants such as Chornobyl (Ukraine, 1986) or Fukushima (Japan, 2011). Investigating the impact of radio-contaminated areas on wildlife arises as a unique opportunity to understand the adaptive capacity of organisms exposed to extreme levels of an abiotic factor that has only been historically present in the Earth at much lower doses (Karam and Leslie, 2005). However, despite these research efforts, there is still no scientific consensus about the long-term effects of ionizing radiation on the ecology and evolution of wildlife in radio-contaminated areas (Beresford *et al.*, 2020).

Amphibians are a great study system to evaluate the ecological and evolutionary effects of radiation. Many species inhabit both terrestrial and aquatic environments, exposing them to a full range of radioactive conditions. Amphibians often have an intermediate life-history pace with one generation every 2 to 3 years in most cases, and their low dispersal capacities facilitate the estimation of absorbed ionizing radiation (Burraco *et al.*, 2021b). Also, amphibian ecophysiology in response to environmental stress is relatively well known (Burraco and Gabor, 2023). Research has extensively examined the life history, physiology and genetics of Eastern tree frogs *Hyla orientalis* (Fig. 1A) living across a wide gradient of radioactive contamination within and around the Chornobyl Exclusion Zone, including their complete exposure to both internal and external radiation (Burraco *et al.*, 2021a, 2023; Burraco and Orizaola, 2022; Car *et al.*, 2022, 2023). Overall, studies on Chornobyl tree frogs have revealed that three decades after the accident, the levels of radioactive isotopes accumulated (i.e. total absorbed dose rate) by frogs currently living in the area are below those considered as potentially dangerous by international standards (ICRP, 2008; Burraco *et al.*, 2021b). This likely explains the lack of impact of radiation on different physiological traits in Chornobyl frogs, including liver morphology associated with organ function and individual health (Burraco *et al.*, 2023), blood biochemistry (Burraco *et al.*, 2021a), lifespan, the stress hormone corticosterone or the ageing marker telomere length (Burraco *et al.*, 2024). Another study found that Chornobyl tree frogs are much darker within the Exclusion Zone compared to those in the surrounding control area (44% darker, on average; Burraco and Orizaola, 2022). Differences in skin coloration of frogs were linked to high radiation levels at the time of the accident; frogs collected from areas with the highest levels of radiation in 1986 were darker than frogs from areas than those that experienced lower levels of radiation (Burraco and Orizaola, 2022). This darker pigmentation (i.e. higher concentration of melanin) detected in Chornobyl frogs still nowadays could be a consequence of rapid evolution linked to the protective role of melanin against ionizing radiation as found in organisms with lower level of organization (Cordero and Casadevall, 2020).

Beyond the eco-evolutionary significance of the study of ionizing radiation, understanding its long-term effects is crucial for accurately assessing the impact of nuclear accidents on wildlife (Beresford *et al.*, 2020). A comprehensive evaluation of the consequences of extreme environments, such as those contaminated with ionizing radiation, requires integrating physiological and genetic information with a solid understanding of species biology. This knowledge is also relevant for assessing the level of risk to human repopulation of these areas. Field research in radiocontaminated areas should be complemented with additional approaches to investigate whether the effects of radiation are isotope-dependent or time (or dose-)-dependent, how these effects may change throughout the life cycle of different organisms and whether they include transgenerational consequences.

## Relevance of Eco-Physiology for Wildlife Conservation

Research on the impact of extreme environments—both natural and anthropogenic—on wildlife can contribute to a better understanding of the responses and adaptations to environmental variation. This may be particularly helpful in the current scenario of rapid environmental change. The study of eco-evolutionary responses in extreme environments requires, at least, previously acquiring the following knowledge: (i) the range for the studied environmental condition and population or species, including historic exposure to the studied conditions; (ii) the basics of the species biology such as generation time, phenotypic plasticity and physiological levels under common ranges of variation of the studied conditions; (iii) possible interactions between the main studied condition and other relevant factors. Although not easy to acquire, this information will allow investigating whether wild populations have evolved specific physiological adaptations to cope with extreme environmental conditions.

During the last decades, ecophysiologists have increasingly highlighted the link between various physiological mechanisms and how environmental conditions impact animal health, finally shaping eco-evolutionary dynamics (Madliger *et al.*, 2018; Isaksson, 2020). Some mechanisms have been presented as good indicators of wildlife responses and adaptations to stressful conditions. This is the case, among others, of organismal and tissue-specific metabolism, glucocorticoids (e.g. cortisol, corticosterone), heat shock proteins (HSPs), oxidative stress indicators, immune-related genes (immunoglobulins, cytokines) or telomere length (Sørensen *et al.*, 2003; McCormick and Romero, 2017; Koch *et al.*, 2021; Ohmer *et al.*, 2021; Costantini, 2024). Such integrated stress response often involves energy costly process to restore homeostasis (McEwen and Wingfield, 2003), which can finally result in fitness decreases. For instance, glucocorticoids can modulate mitochondrial activity and the antioxidant machinery, whereas the overproduction of free radicals can influence cytokine signalling pathways and damage essential cell structures such as lipids, proteins or DNA (Vitale *et al.*, 2013; Cain and Cidlowski, 2017). Markers, such as HSPs, greatly respond to specific challenging conditions like thermal stress, enabling researchers to evaluate species' adaptability to climate change (Sørensen *et al.*, 2003). Other markers, such as telomere length, are considered as crucial biomarkers for the cumulative impact of environmental stress on cellular and organismal ageing and health (Angelier *et al.*, 2018; Burraco *et al.*, 2020; Chatelain *et al.*, 2020; Salmón and Burraco, 2022). Indeed, telomere dynamics are thought to be associated with high release of glucocorticoids, oxidative stress, heat shock proteins or the immune function, which likely explains the link between telomere shortening and accelerated organismal ageing or reduced health span (López-Otín *et al.*, 2023). The use of epigenetic markers, and particularly the epigenetic clocks, also seems to be a prominent tool to comprehensibly understand how environmental stressors influence both immediate and long-term health outcomes (Rey *et al.*, 2020; Le Clercq *et al.*, 2023).

Establishing baseline data on ecophysiological markers helps evolutionary ecologists and conservationists to identify deviations from normal health states when conditions become extreme. This includes the understanding of daily and seasonal variation and thresholds beyond physiological levels that become detrimental for the organism. An accurate evaluation of animal health under extreme environments should include both field and laboratory longitudinal approaches measuring different physiological markers, complemented with information on life-history traits (e.g. growth, reproduction), ideally throughout the lifetime of a number of individuals of populations exposed to a range of natural and anthropogenic environmental conditions (Fig. 1B, left). By comparing these responses under standard versus extreme conditions, we can identify shifts that may be adaptive or maladaptive, and begin to elucidate the underlying mechanisms driving population resilience or decline (Fig. 1B, middle). Physiological markers, in this context, offer valuable insight into the sub-lethal impacts of environmental stressors and can serve as early-warning indicators of population health.

Importantly, once such biomarkers are validated, they can be integrated into mechanistic frameworks such as Species Distribution Models (SDMs; Fig. 1B, right) to forecast species distribution under future scenarios (Kearney and Porter, 2009; Johnston *et al.*, 2019; e.g. Becker *et al.*, 2020; Burraco *et al.*, 2021d). These models are a promising tool to inform conservation actions by identifying vulnerable populations which, therefore, should include the development of targeted management and restoration strategies. In addition, advances in technologies, such as high-throughput screenings (e.g. metabolomics, proteomics) or remote sensing monitoring, in combination with currently available physiological and modelling tools, could greatly help to anticipate the effects of global changes and particularly of extreme events, on the demography and health of wild populations.
